# Production of extracellular amylase contributes to the colonization of *Bacillus cereus* 0–9 in wheat roots

**DOI:** 10.1186/s12866-022-02618-7

**Published:** 2022-08-22

**Authors:** Qiubin Huang, Huiping Liu, Juanmei Zhang, Shaowei Wang, Fengying Liu, Chengdie Li, Gang Wang

**Affiliations:** 1 Institute of Microbial Engineering, Henan University, Kaifeng, Henan 475004 People’s Republic of China; 2 School of Life Sciences, Henan University, Kaifeng, Henan 475004 People’s Republic of China; 3 Pharmaceutical College, Henan University, Kaifeng, Henan 475004 People’s Republic of China

**Keywords:** Extracellular amylase, *Bacillus cereus*, Colonization, Biocontrol ability

## Abstract

**Background:**

Bacteria usually secrete a variety of extracellular enzymes to degrade extracellular macromolecules to meet their nutritional needs and enhance their environmental adaptability. *Bacillus cereus* 0–9, a biocontrol bacterial strain isolated from wheat roots, has three genes annotated as encoding amylases in the genome, but their functions are unknown, and whether they are involved in the colonization process of the bacterium remains to be further studied.

**Methods:**

Mutant gene strains and fluorescently tagged strains were constructed by homologous recombination, and amylase protein was expressed in the prokaryotic *Escherichia coli* BL21(DE3) expression system. The iodine staining method was used to measure the activity of amylase proteins. We further observed the colonization abilities of the test strains in wheat roots through frozen section technology.

**Results:**

The results showed that there were three amylase-encoding genes, *amyC*, *amyP* and *amyS,* in the *B. cereus* 0–9 genome. Among the three amylase encoding genes, only *amyS* produced extracellular amylase whose secretion was related to signal peptide at position 1–27. The AmyS protein encoded by the *amyS* gene is an α-amylase. The growth of *Rhizoctonia cerealis* was inhibited 84.7% by *B. cereus* 0–9, but the biocontrol ability of the Δ*amyS* strain decreased to 43.8% and that of Δ*amyS*/*amyS* was restored when the *amyS* gene was complemented. Furthermore, the biocontrol ability of the Δ*amySec* strain was decreased to 46.8%, almost the same as that of the Δ*amyS* mutant. Due to the deletion of the *amyS* gene, the colonization capacities of Δ*amyS* (RFP) and Δ*amySec* (RFP) in wheat roots decreased, while that of Δ*amyS*/*amyS* (RFP) was restored after the *amyS* gene was complemented, indicating that the *amyS* gene influences the colonization of *B. cereus 0–9* in wheat roots. In addition, the colonization and biocontrol abilities of the mutant were restored after the addition of sugars, such as glucose and maltose.

**Conclusions:**

*B. cereus* 0–9 encodes three genes annotated as amylases, *amyC*, *amyP* and *amyS*. Only the deletion of the *amyS* gene with a signal peptide did not produce extracellular amylase. The AmyS protein encoded by the *amyS* gene is an α-amylase. Our results indicated that the *amyS* gene is closely related to the colonization abilities of *B. cereus* 0–9 in wheat roots and the biocontrol abilities of *B. cereus* 0–9 to fight against *R. cerealis*. The extracellular amylase produced by *B. cereus* 0–9 can hydrolyze starch and use glucose, maltose and other nutrients to meet the needs of bacterial growth. Therefore, it is very possible that the secretion and hydrolytic activities of extracellular amylase can promote the colonization of *B. cereus* 0–9 in wheat roots and play important roles in the prevention and control of plant diseases. Our results contribute to exploring the mechanisms of microbial colonization in plant roots.

**Supplementary Information:**

The online version contains supplementary material available at 10.1186/s12866-022-02618-7.

## Introduction

Wheat sharp-eyespot caused by the plant pathogenic fungus *Rhizoctonia cerealis* is an important soil-borne disease widely distributed in wheat-producing areas in China [1, 2]. Currently, there is a lack of effective wheat varieties resistant to sharp eyespot, and the disease is mainly prevented by chemical pesticides. However, the frequent use of chemical pesticides not only leads to environmental pollution but may also result in the selection of resistant microorganisms [3, 4]. As an alternative disease control strategy, biocontrol using rhizosphere microorganisms and their metabolites has gradually attracted strong attention from researchers worldwide [5–8]. It has been reported that *Bacillus*, *Pseudomonas* and *Trichoderma* have biocontrol activities and can be used for the biocontrol of wheat sharp-eyespot disease [9–12]. These microorganisms inhibit the survival of pathogens in plant roots mainly through competition, antibiosis and superparasitism [10, 13]. Compared with chemical control, the disadvantage of microbial biocontrol is that the control effect is greatly influenced by environmental factors and is unstable [1, 2]. Microorganism survival is easily affected by temperature, humidity, availability of nutrients, and competition by indigenous microorganisms [13, 14]. Therefore, the efficient colonization of biocontrol microorganisms in plant roots is a prerequisite for their biocontrol effects [15–17].

In the case of nutrient deficiency, many kinds of bacteria produce and secrete various degrading enzymes, such as amylases, proteases, and phospholipases, at certain growth stages. Bacteria use these enzymes to degrade macromolecular substances outside the cells to obtain nutrients to meet their nutritional needs and improve their adaptability to the environment [18, 19]. Therefore, whether the ability of biocontrol bacteria to produce extracellular degrading enzymes under certain conditions is involved in their colonization process remains to be further studied.

Amylase is a general term for enzymes that hydrolyze starch and glycogen. According to their different substrates, amylases can be divided into α-amylases and β-amylases. The former hydrolyze amylose, and the latter hydrolyze amylopectin. Extracellular amylases often have signal peptides at the N-terminus, which are synthesized inside the cells and then secreted outside by the Sec protein secretion system [20–24]. To date, there have been many studies on *Bacillus amyloliquefaciens* in the bacterial colonization of the host [25–29]. However, it is rarely reported whether the production of amylase is related to the colonization and adaptability of *Bacillus cereus*.

*B. cereus* strain have the characteristics of fast growth rate, large spore production, strong stress resistance, and rapid colonization on the surface of plants, so they have good application prospects [7, 10]. *B. cereus* 0–9, a bacterium isolated from wheat roots, has biocontrol potential against wheat sharp eyespot and the capacity to secrete extracellular amylase. Previous studies have shown that the phosphotransferase system (PTS) of this bacterium is involved in glucose absorption and rhizosphere colonization capacity and that the bacterial colonization capacity in the rhizosphere is related to its biocontrol activity [9]. Therefore, this study aims to analyze whether extracellular amylase affects the colonization ability of *B. cereus* 0–9 in the roots of wheat and its biocontrol ability against wheat sharp eyespot disease.

## Materials and methods

### Microorganisms, plasmids and growth conditions

The plant pathogenic fungi, bacterial strains and plasmids used in this work are listed in Table 1 [30, 31]. The test strains of wild-type *B. cereus* 0–9 and its derivatives were cultured aerobically in Luria–Bertani (LB) medium at 30 °C [9]. *Escherichia coli* 116 cells were grown in LB medium for the propagation of plasmids for DNA extraction. *E. coli* GM2163 was grown in a LB medium for the propagation of plasmids without methylation. *R. cerealis* strain (Table 1) was cultured on potato dextrose agar (PDA) for biocontrol assays. Plasmid pMAD (Table 1), containing *lacZ* gene, ampicillin and erythromycin resistance genes, was used for generating gene inactivation mutants. Plasmid pAD123-Pgal (Table 1), containing ampicillin and chloramphenicol resistance genes, was used for gene complementation [8]. When needed, antibiotics were added at the final concentrations as follows: ampicillin 100 μg/mL, chloramphenicol 20 μg/mL, erythromycin 3 μg/mL and kanamycin 50 μg/mL.

**Table 1:** *B. cereus* strains, fungus and the plasmids used in this study

Name	Property
*B. cereus* 0–9	Endophytic bacteria isolated from wheat roots by us
Δ*amyC*	The *amyC* gene mutant strain of *B. cereus* 0–9
Δ*amyP*	The *amyP* gene mutant strain of *B. cereus* 0–9
Δ*amyS*	The *amyS* gene mutant strain of *B. cereus* 0–9
Δ*amySec*	Mutant strain of *B. cereus* 0–9 with deletion of only the signal peptide coding region of the amylase gene
Δ*amyS/amyS*	The complementary strain of Δ*amyS*
*B. cereus* 0–9 (RFP)	The red fluorescent protein label strain of *B. cereus* 0–9
Δ*amyS* (RFP)	The red fluorescent protein label strain of Δ*amyS*
Δ*amyS/amyS* (RFP)	The red fluorescent protein label strain of Δ*amyS/amyS*
Δ*amySec* (RFP)	The red fluorescent protein label strain of Δ*amySec*
*E.coli* BL21/pET28a-*amyS*	Strain expressing *amyS* without a signal peptide coding region
*R. cerealis*	Fungus causing wheat sharp-eyespot
Plasmid pMAD	Plasmid containing lacZ, Amp and Erm tags and used for the gene mutation process
Plasmid pMADchi	Plasmid containing upstream and downstream fragments of *chi* gene in the polyclonal cleavage site of plasmid pMAD [30]
Plasmid pAD123-Pgal	Plasmid used for the construction of complementation strains, containing Amp and Cm tag [31]
Plasmid pET-28a	Plasmid used for gene expression, containing a km tag

### Bioinformatics analysis

Whole-genome sequencing (GenBank: CP042874.1, CP042875.1, CP042876.1) and subsequent gene function annotation revealed that the *B. cereus* 0–9 genome encodes three Amy protein (Protein ID: QEF15890.1, QEF19997.1, QEF17986.1). The putative amino acid sequences used in this study were downloaded from the GenBank nucleotide sequence database. The signal peptide information was analyzed through SignalP 4.1 software by Detai Biologic (http://www.detaibio.com/tools/signal-peptide.html). A Simple Modular Architecture Research Tool (SMART) software (http://smart.embl-heidelberg.de/smart/change_mode.pl) was used to analyze the PFAM domains.

### Construction of gene deletion and complementation strains

The *B. cereus* 0–9 gene knockout method was described in our previous publication [30]. The primer pair *amyS*-up-*Bam*HI-s (Table S1, 5’-ACACGGATCCACCATCAATTCCACCATTTACA-3’) and *amyS*-up-*Hin*dIII-a (5’-CACAAAGCTTGATCAGTTTCCATATATGTTCA-3’) was used to amplify a fragment upstream of *amyS*. The primer pair *amyS*-down-*Hin*dIII-s (5’-ACACAAGCTTTGTTATTCTTTTAAACATCTG-3’) and *amyS*-down-*Eco*RI-a (5’-CACAGAATTCTCAGTTAGTTTTACAATAAGAG-3’) was used to amplify a fragment downstream of *amyS*. The two fragments were gel-purified, digested with *Hin*dIII, and then ligated with T4 DNA ligase to form a new fragment, *amyS*-AB. This *amyS*-AB fragment was digested with *Bam*HI and *EcoR*I and then ligated with T4 DNA ligase into the *Bam*HI/*EcoR*I site of plasmid pMAD to generate the *amyS* gene deletion vector pMAD-AB. The pMAD-AB vector was transformed into *E. coli* 116-competent cells by thermal transformation at 42 °C for propagation of the plasmid. pMAD-AB was extracted and identified with restriction endonucleases *Bam*HI/*EcoR*I. Subsequently, the plasmid was transformed into *E. coli* GM2163 by electroporation (1700 V, Eporator, Eppendorf) and also extracted and identified with restriction endonucleases *Bam*HI/*EcoR*I. The pMAD-AB plasmid from *E. coli* GM2163 was transformed into *B. cereus* strain 0–9-competent cells by electroporation and cultured on tryptic soy agar (TSA; BD Diagnostics, Sparks, MD) plates supplemented with erythromycin (3 μg/mL) and 5-bromo-4-chloro-3-indolyl-β-D-galactopyranoside (X-gal, 100 μg/mL) at 30 °C overnight. One blue colony was inoculated into 50 mL of TSB medium (BD Diagnostics) supplemented with erythromycin (3 μg/mL) and incubated at 30 °C at 200 r/min overnight. Five hundred microliters of the culture was inoculated into 50 mL of TSB medium in a flask. The flask was incubated at 30 °C at 200 r/min until the optical density of the culture reached 0.01 at 600 nm, and then incubated with shaking for 2 h at 30 °C at 200 r/min followed by 6 h at 42 °C. One hundred microliters of this culture was plated on TSA agar plates containing erythromycin (3 μg/mL) and X-gal (100 μg/mL) and incubated at 42 °C for 48 h. One blue colony was inoculated into 50 mL of TSB medium supplemented with erythromycin (3 μg/mL) and incubated at 30 °C and 200 r/min overnight. One hundred microliters of this culture was plated on TSA agar plates containing X-gal (100 μg/mL) and incubated at 30 °C overnight. The white colonies were isolated on the same medium at 30 °C and verified for erythromycin sensitivity. To confirm the gene deletion, chromosomal DNA was extracted from two candidate clones and used in PCR with the *amyS*-up-*Bam*HI-s and *amyS*-down-*Eco*RI-a primer pair [8]. The same method was used to make ∆*amyC* and *∆amyP*.

Moreover, a DNA fragment containing the *amyS*-coding region of *B. cereus* 0–9 and its native promoter was cloned into pAD123-Pgal (Table S1) at the *Bam*HI and *Xho*I sites to construct the recombinant plasmid pAD123-Pgal-*amyS*_pro_. Then, the recombinant plasmid pAD123-Pgal-*amyS*_pro_ was transformed into competent cells of the *∆amyS* mutant to construct the reverse complementation strain ∆*amyS/amyS*. Meanwhile, the primer pair *amySec*-*Bam*HI-s and *amyS*-*Xho*I-a was used to amplify a fragment containing the *B. cereus* 0–9 *amyS*-encoding region and its native promoter but without a signal peptide. Then, the fragment was digested with *Bam*HI and *Xho*I and ligated with T4 DNA ligase into the *Bam*HI/*Xho*I site of pAD123-Pgal to generate the vector pAD123-*Pgal*-*amySec*. The recombinant plasmid was transformed into the ∆*amyS* mutant to obtain the amylase signal peptide gene deletion mutant ∆*amySec*. The only difference between strains ∆*amyS/amyS* and ∆*amySec* is the presence of a signal peptide. The *mCherry* gene encoding red fluorescent protein was cloned into pMADchi at the *Bam*HI and *Xho*I sites to construct the recombinant plasmid pMADchi-*mCherry*, which was used to construct the labeled strain by allelic exchange. The same method was also used to create *B. cereus* 0–9 (RFP), ∆*amyS* (RFP), ∆*amyS/amyS* (RFP) and ∆*amySec* (RFP).

### Purification of the AmyS protein and determination of its enzyme activity

Using the genome of wild-type *B. cereus* 0–9 as a template, the *amyS* ORF without a signal peptide coding region was amplified and ligated into the pET28a vector with *Bam*HI and *Xho*I restriction sites to construct the recombinant vector, which was transferred into the *E. coli* BL21(DE3) strain to obtain the *amyS* expression strain *E. coli* BL21(DE3)/pET28a-*amyS*. The strain was cultured in liquid LB medium containing 100 μg/mL ampicillin at 37 °C and 200 r/min for 6 h. Then, the bacterial suspension at a concentration of 10^5^ cfu/mL was supplemented with 100 μmol/L isopropyl β-D-thiogalactoside (IPTG) for induction culture at 22 °C and 200 r/min for 6 h, followed by centrifugation at 13,201 × g. Then, bacteria were resuspended in 60 mL lysis buffer containing 20 mM Tris/HCl (pH 8.0), 50 mM NaCl and 5 mM 2-mercaptoethanol and subjected to ultrasonication in ice water for 30 min. A 20 mM imidazole solution was used for Ni column purification, and the purified protein was obtained [31]. Protease activity was determined by the plate iodine fumigation method [32, 33].

### Determination of the extracellular amylase activity of *B. cereus* 0–9 and deletion strains

*B. cereus* 0–9 and its mutants were inoculated in LB medium and cultured at 30 °C for 16 h to a bacterial suspension concentration of 10^8^ cfu/mL, followed by centrifugation at 13,201 × g. The bacteria were collected, suspended in phosphate-buffered saline (PBS), centrifuged at 13,201 × g and washed twice with PBS. After the supernatant was discarded, an equal volume of sterile water was added to the pellet, and 5 μL of bacterial suspension was harvested, inoculated on LB solid medium containing 1% starch (w/v), cultured at 30 °C for 2 d and fumigated with iodine for 5 min. The consumption of starch was calculated using the hydrolytic circle diameter, and the activity of amylase was determined by the blue value method. The amount (mg) of soluble starch hydrolyzed by 1 mL of enzyme at 65 °C and pH 6.5 for 1 min was defined as one unit of amylase activity (U/mL) [34].

### Determination of the colonization capacity and biocontrol ability of strains

The RFP-labeled strains were inoculated in 100 mL of liquid LB medium and cultured at 30 °C and 200 r/min to obtain a bacterial suspension concentration of 10^8^ cfu/mL, followed by centrifugation at 6,000 × g for 10 min. After the supernatant suspension was discarded, 40 mL of sterile water was added and mixed gently to prepare the bacterial suspension. Fifty germinated wheat seeds (BN AK-58, Purchased from Henan Zhongzhong Lianfeng Seed Industry Co., Ltd., Kaifeng City, China) were added to the bacterial suspension and soaked for 3 h. Then, 5 soaked wheat seeds were placed in a test tube containing sterile sand, with 10 replicates for each strain. Eight milliliters of each strains which including wild-type strain 0–9 or the mutant strains suspended in sterile water after centrifugation was added to each test tube, with sterile water as a blank control. On the basis of the addition of the bacterial suspension, 1 mL of 1% starch solution was added as a treatment group. The other five treatment groups also followed the same method: adding glucose, adding maltose, adding maltodextrin, adding 1% starch and AmyS protein, and adding 1% starch and commercial α-amylase. Finally, the wheat was covered with soil containing fungus *R. cerealis*, placed in a sterile incubator and cultivated under 22 °C with light. After 7 d, fresh roots of wheat were collected and gently washed with water, and the incidence of wheat sharp eyespot in basal stems was assessed according to the disease grading scale of wheat sharp eyespot. Moreover, the disease index of wheat sharp eyespot and biocontrol ability of the tested strains were calculated [35, 36]:

$$\mathrm{Disease}\;\mathrm{index}=\frac{\sum(A\ast B)}{50\ast5}\ast100\%$$

Biocontrol ability = (control disease index—treatment of disease index)/control disease index × 100%

A represents the number of diseased plants in each treatment; B represents the numerical value of the wheat disease grade; 50 represents the total number of wheat seedlings assessed; and 5 represents the numerical value of the highest level of wheat disease.

Subsequently, fresh wheat roots were washed gently with water and observed under a fluorescence microscope (Leica DM4000, Germany). Frozen section technology was used to observe the colonization of RFP label strains in wheat roots [37, 38]. Moreover, the wheat roots were sterilized with 70% ethanol for 30 s followed by 3% NaClO for 30 s and subsequently washed several times with sterilized distilled water. Then, the root samples were ground, diluted and coated on sterilized LB agar plates to determine the colonization capacity of bacteria in wheat roots (CFU/mL/g) [39, 40].

### Statistical evaluations

The colonization capacity and biocontrol ability of the wild-type and mutant strains were generated by plotting the average outcomes of three experiments for each strain. We used the SAS 8.0 tool to analyze the differences in the colonization capacity and biocontrol by one-way analysis of variance (ANOVA) followed by Tukey’s pairwise post hoc comparisons.

## Results

### Bioinformatics analysis of amylase-encoding genes in *B. cereus* 0–9

**Fig. 1:**
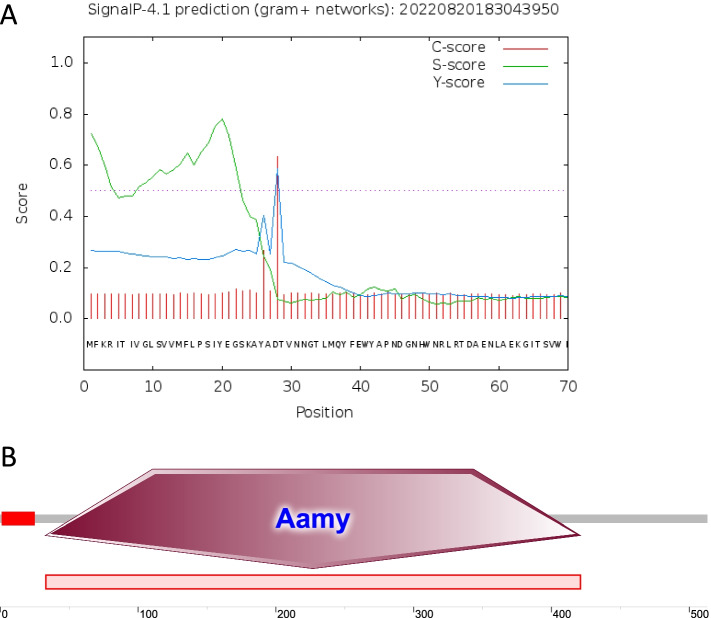
Analysis of the *amyS* gene (FRY47_17030) signal peptide and smart domain. **A** Analysis of the AmyS protein signal peptide through SignalP 4.1. S-score: Each amino acid corresponds to 1 S value, with a high S value in the signal peptide region. C-score: Each amino acid will have a C value, with the highest C value at the shear site. Y-score: The Y value is a parameter considering the S and C values and is more accurate than the C value alone because there may be more than one higher C value in a series but only one shear site; the shear site has a steep S value and a high C value. **B** Analysis of the AmyS protein smart domain using SMART software. Positions 33–421 represent the α-amylase domain, which catalyzes the hydrolysis of (1–4)-α-D-glucosidic linkages in polysaccharides to remove successive α-maltose residues from the nonreducing ends of the chains in the conversion of starch to maltose

We analyzed the *B. cereus* 0–9 genome and found the three amylase-encoding genes, *amyC* (FRY47_05825), *amyP* (FRY47_27030) and *amyS* (FRY47_17030). Among the three Amy proteins, only the AmyS protein contains both a signal peptide and an amylase domain (Fig. 1, Figs. S1 and S2). It is speculated that AmyS may be involved in the extracellular secretion of amylase and the process of starch hydrolysis [41–43].

### Deletion of *amyS *abolishes the ability of *B. cereus* 0–9 to hydrolyze starch

**Fig. 2:**
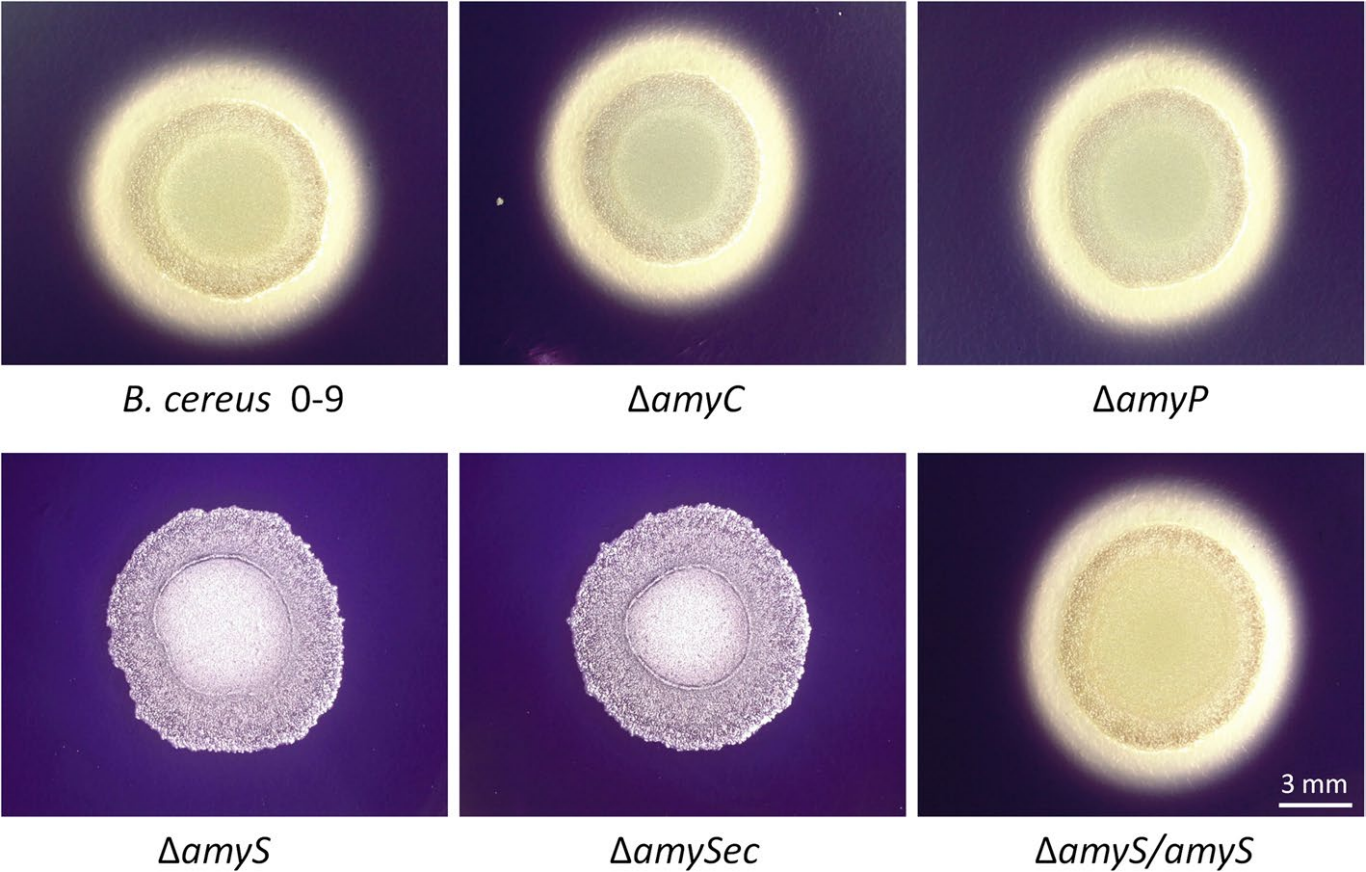
Determination of the capacity of *B.cereus* 0–9, Δ*amyS*, Δ*amyC*, Δ*amyP*, Δ*amyS*/*amyS* and Δ*amySec* strains to hydrolyze starch. The size of the white transparent circle represents the ability of the strain to produce extracellular enzymes

We constructed Δ*amyS*, Δ*amyC* and Δ*amyP* amylase mutants and analyzed their abilities to hydrolyze extracellular starch on LB plates (Fig. 2). Unlike Δ*amyC* and Δ*amyP* mutant, Δ*amyS* mutant could not form a transparent circle on the LB starch plates, suggesting that *B. cereus* 0–9 ability to hydrolyze starch was lost in the absence of AmyS. Complementation of Δ*amyS* strain with native *amyS* gene (Δ*amyS*/*amyS*) partially restored the hydrolysis activity of *B. cereus* 0–9 on the LB starch plates. However, complementation of ΔamyS with *amyS* gene lacking signal peptide (Δ*amySec*) did not restore the starch hydrolysis activity of *B. cereus* 0–9 (Fig. 2). The results showed that the signal peptide of the AmyS protein is critical for the starch hydrolysis ability of *B. cereus* 0–9.

### AmyS protein is an α-amylase

**Fig. 3:**
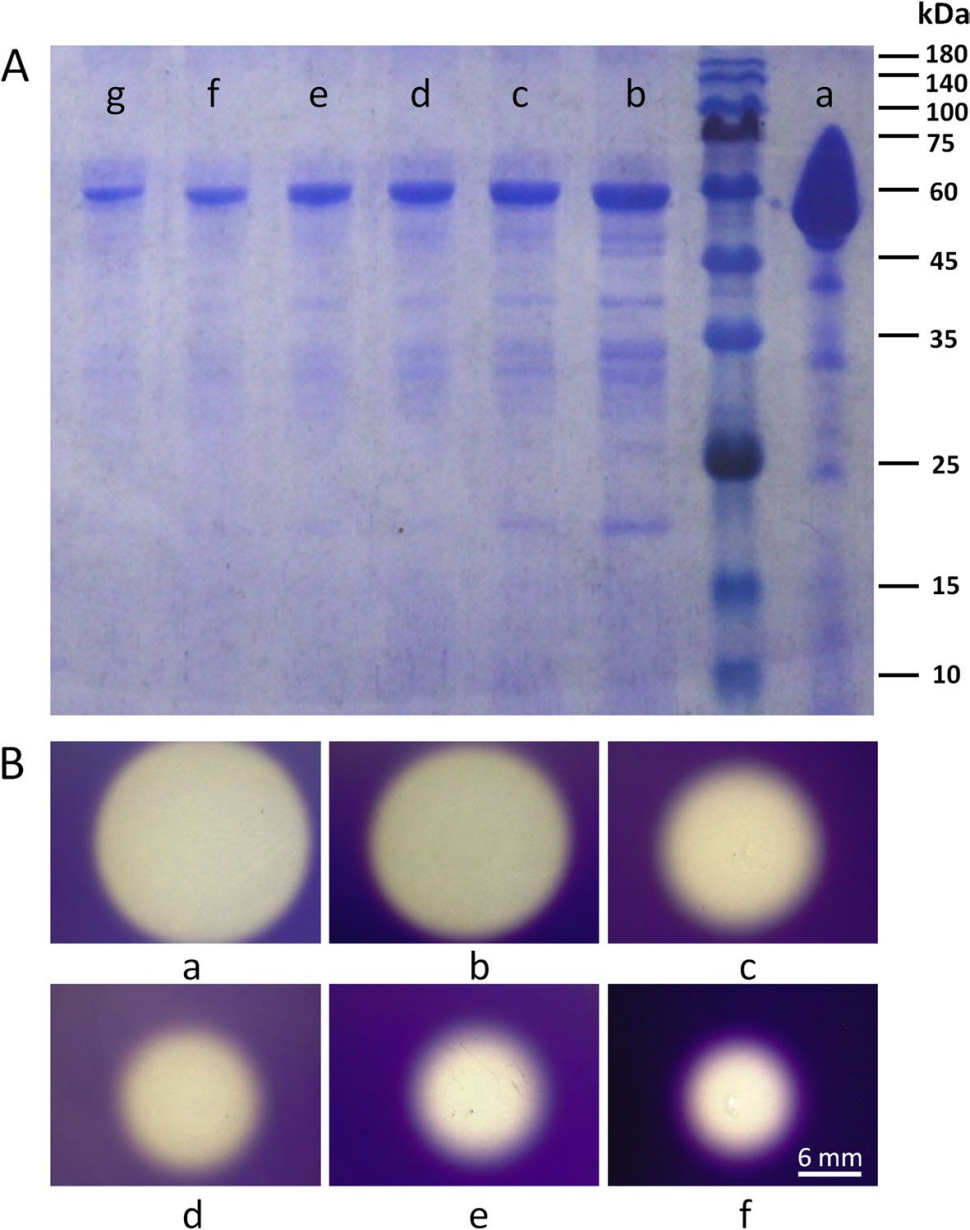
(**A**) Induced expression of the *amyS* gene in the *E. coli* BL21(DE3) strain. **a** represents commercial α-amylase (Ruibio, Germany); (**b**) represents unpurified AmyS protein; (**c**) represents purified AmyS protein; (**d**) represents purified protein diluted twice; (**e**) represents purified protein diluted threefold; (**f**) represents purified protein diluted fourfold; (**g**) represents purified protein diluted fivefold. **B** Determination of the enzymatic activity of AmyS protein. Five microliters of AmyS protein with different concentrations was harvested, inoculated on LB solid medium containing 1% starch (w/v), cultured at 30 °C for 2 d and fumigated with iodine for 5 min. The size of the white transparent circle represents the enzymatic activity of the AmyS protein. **a** represents the commercial α-amylase; (**b**) represents purified AmyS protein; (**c**) represents the purified protein diluted twice; (**d**) represents the purified protein diluted threefold; (**e**) represents the purified protein diluted fourfold; (**f**) represents the purified protein diluted fivefold

We found that the function of the *amyS* gene is related to the starch hydrolysis ability of *B. cereus* 0–9, but whether the *amyS* gene encodes α-amylase has not been determined*.* We purified AmyS as a 55 kDa His-tagged protein in *E.coli* BL21/pET28a-*amyS* (Fig. 3A), and its amylase activity was detected by the blue value method in LB medium containing 1% starch. The AmyS protein produced a transparent circle that was similar to that obtained with commercial amylase (Fig. 3B). These results indicate that the AmyS protein encoded by the *amyS* gene is an α-amylase.

### AmyS is involved in the ability of *B. cereus* 0–9 to fight against *R. cerealis*

**Fig. 4:**
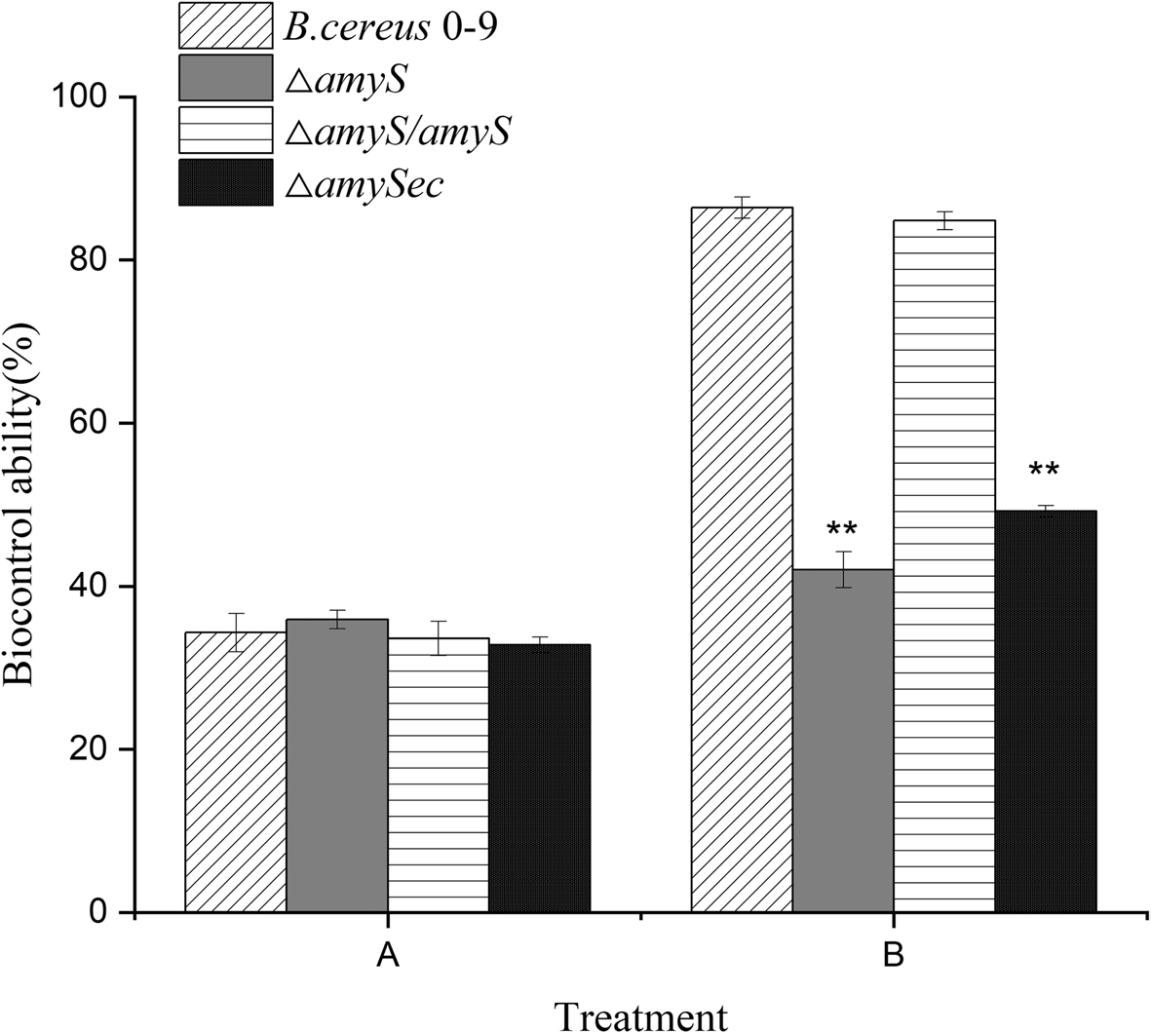
Biocontrol abilities of the *B.cereus* 0–9, Δ*amyS*, Δ*amyS*/*amyS* and Δ*amySec* strains on wheat sharp eyespot. Fresh wheat roots were assessed according to the disease grading scale of wheat sharp eyespot, and the biocontrol abilities of the tested strains were calculated. The letter A stands for biocontrol capacity without the test strains; the letter B represents biocontrol capacity of the test strains; the symbol “**” indicates that the *P* value of the difference between the data of *B. cereus* 0–9 and the mutants was extremely significant (*P* < 0.01)

To validate whether AmyS is related to the biocontrol ability of *B. cereus* 0–9, we determined the activities of the Δ*amyS*, Δ*amyS*/*amyS* and Δ*amySec* strains against *R. cerealis*. The result showed that the biocontrol capacity of the roots were very weak without bacterial suspension added (Fig. 4A). With the addition of *B.cereus* 0–9 and the derivative strains, the biocontrol capacity of the roots was improved, but the effect was different. The Δ*amyS* strain has a lower biocontrol activity (43.8%) compared to its parental strain (84.7%). The parental strain biocontrol activity was restored in Δ*amyS*/*amyS* strain. Interestingly, the biocontrol activity of the Δ*amySec* strain was almost the same as that of the Δ*amyS* strain (Fig. 4B). This result indicates that AmyS contributes to the biological control of *B. cereus* 0–9, and its contribution depends on the presence of the signal peptide.

### Extracellular amylase affects the colonization capacity of *B. cereus* 0–9

**Fig. 5:**
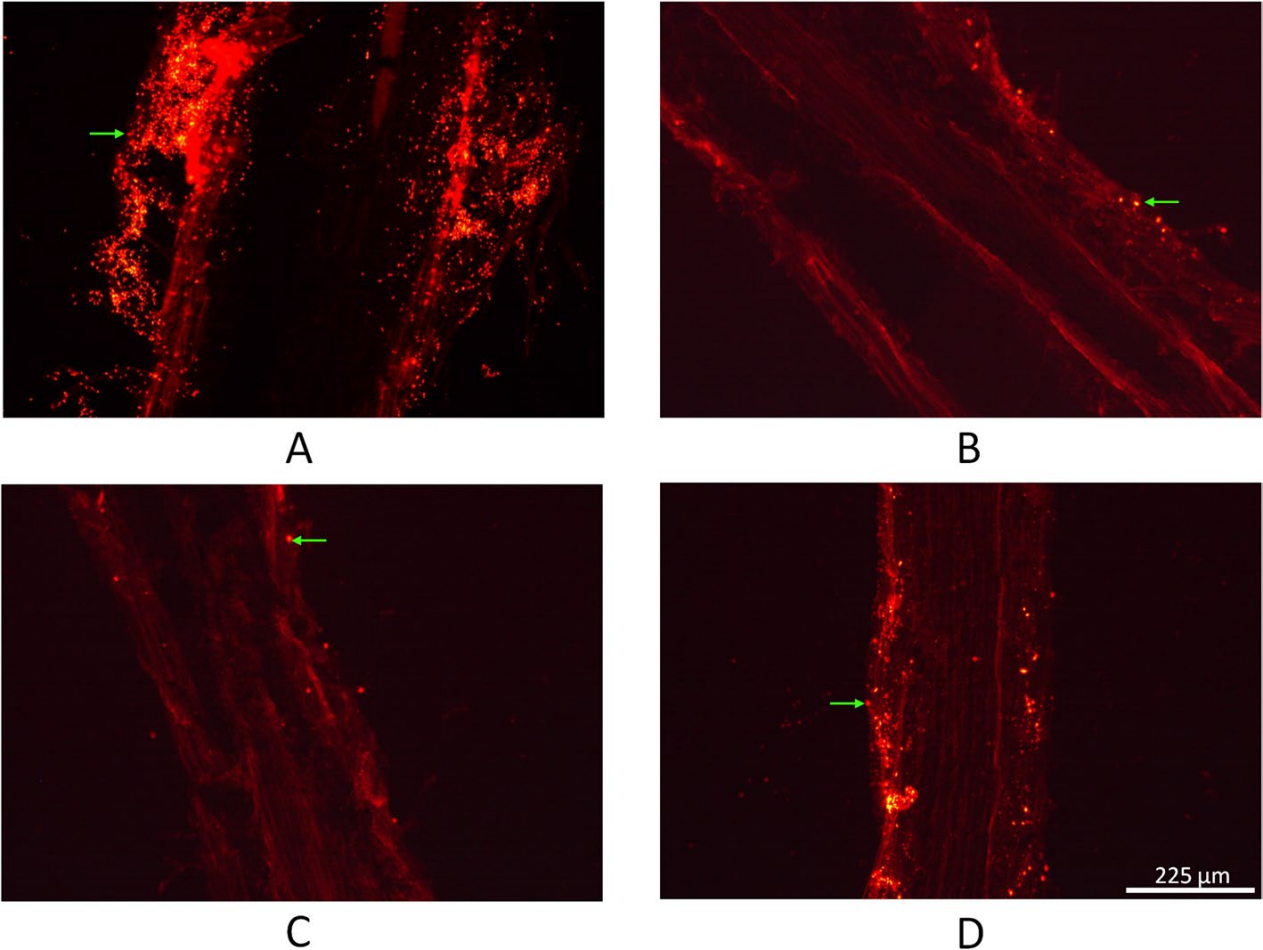
Determination of the colonization capacities of the *B.cereus* 0–9 (RFP), Δ*amyS* (RFP), Δ*amyS*/*amyS* (RFP) and Δ*amySec* (RFP) strains. Frozen section technology was used to observe the colonization of RFP label strains in wheat roots. **A** The colonization capacity of *B. cereus* 0–9 (RFP); (**B**) the colonization capacity of the Δ*amyS* (RFP) strain; (**C**) the colonization capacity of the Δ*amyS*ec (RFP) strain; (**D**) the colonization capacity of the Δ*amyS/amyS* (RFP) strain. The green arrow (→ or ←) represents the RFP-labeled bacteria

To determine the colonization capacity of the WT, Δ*amyS*, Δ*amyS*/*amyS* and Δ*amySec* strains, wheat roots were inoculated with the RFP-labeled strains, Δ*amyS* (RFP), Δ*amyS*/*amyS* (RFP) and Δ*amySec* (RFP) (Fig. 5). After 7 days, we could not observe fluorescent single rod-shaped cells and clusters in the root of the negative control treatment (no inoculation with RFP strains, Fig. S3). However, we could observe single rod-shaped cells and clusters in wheat roots inoculated with RFP-labeled strains. Figure 5 shows that the Δ*amyS* (RFP) and Δ*amySec* (RFP) strains (Figs. 5B and C) had a lower colonization ability compared to WT (Fig. 5A), and Δ*amyS*/*amyS* (RFP) strain (Fig. 5D), indicating that the *amyS* gene influences the colonization of *B. cereus* 0–9 in wheat roots.

**Fig. 6:**
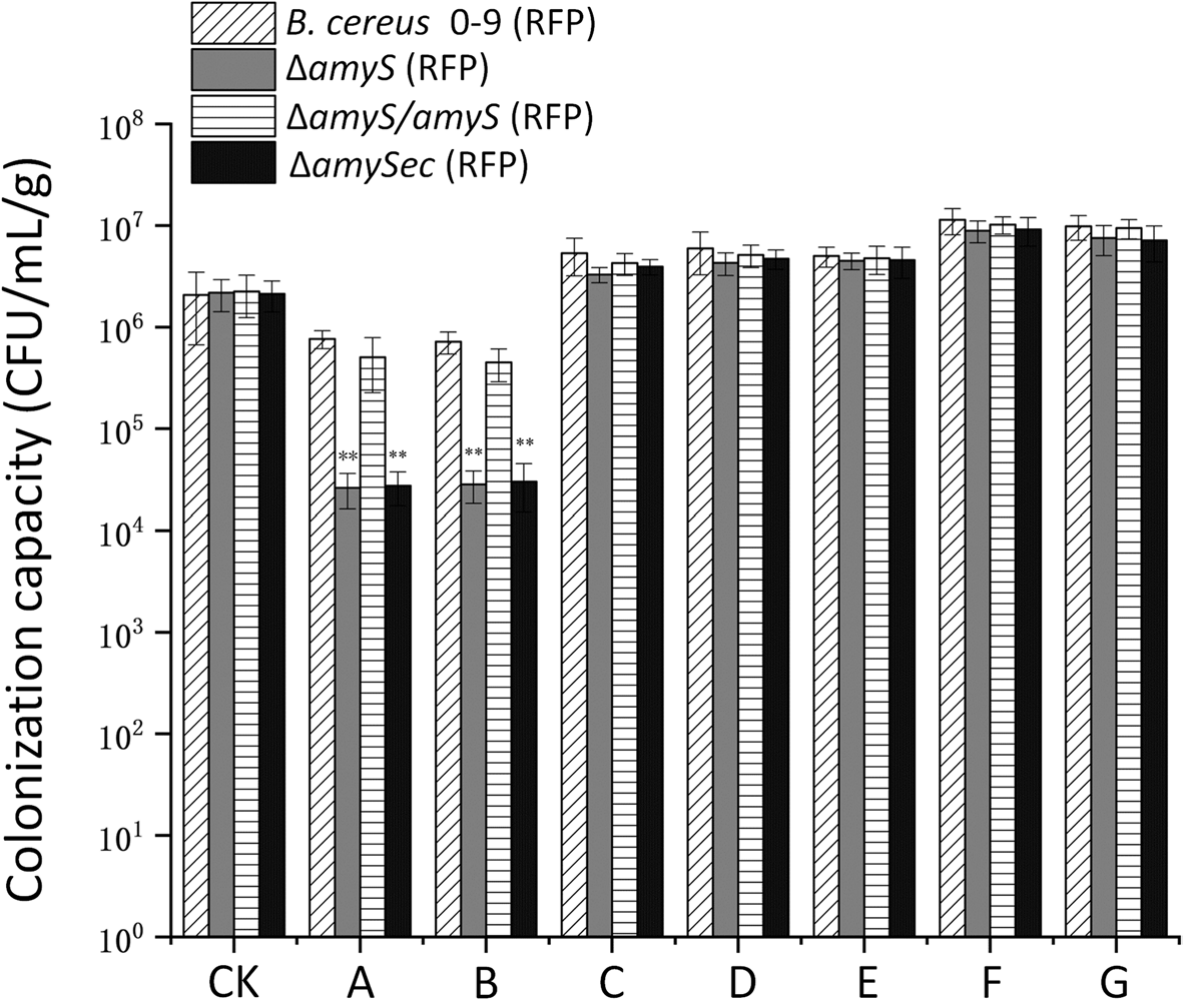
Colonization of wheat roots by the *B.cereus* 0–9 (RFP), Δ*amyS* (RFP), Δ*amyS*/*amyS* (RFP) and Δ*amySec* (RFP) strains. (CK) represents initial bacterial counts; (**A**) represents the colonization capacity of the strains after 7 d; (**B**) represents exogenous starch; (**C**) represents exogenous glucose; (**D**) represents exogenous maltose; (**E**) represents exogenous maltodextrin; (**F**) represents exogenous starch and commercial α-amylase; (**G**) represents exogenous starch and AmyS protein. The symbol “**” indicates that the *P* value of the difference between the data of *B. cereus* 0–9 and the mutants was extremely significant (*P* < 0.01)

The colonization ability of bacteria in plant roots is related to the nutrient acquisition of bacteria, and it is also an important manifestation of bacterial adaptability. It is well known that amylase can hydrolyze starch to produce glucose, maltose, maltodextrin and other substances [21, 22], while wheat seeds contain a large amount of starch; therefore, we studied the changes in the colonization ability of *B. cereus* 0–9 after adding these substances. The results showed that the colonization abilities of the four strains in wheat roots were significantly improved after adding 0.1% glucose, 0.1% maltose or 0.1% maltodextrin. Exogenous addition of starch alone did not cause a change in the bacterial colonization ability of the four strains, while addition of both exogenous starch and AmyS protein (with commercial α-amylase as a control) enhanced their colonization capacity. Interestingly, the colonization abilities of the Δ*amyS* (RFP) and Δ*amySec* (RFP) mutants were restored to wild-type levels in the presence of both extracellular starch and AmyS (Fig. 6). These results demonstrated that starch hydrolysis products such as glucose, maltose and maltodextrin can be used for the growth.

### Starch hydrolysate contributes to the biocontrol ability of *B. cereus* 0–9

To explore the effect of starch hydrolysate on the biocontrol ability of *B. cereus* 0–9, we added starch hydrolysate during the biocontrol experiment and measured the disease index and biocontrol effect of the WT, Δ*amyS*, Δ*amyS*/*amyS* and Δ*amySec* strains. The results showed that after the addition of glucose, the wheat sharp eyespot index decreased, and the biocontrol effects of the strains improved from 84.7% to 87.9%, the treatment with added exogenous maltose and maltodextrin also improved (86.1% and 86.7%). The biocontrol abilities of mutant strains Δ*amyS* and Δ*amySec* reached 72.3% and 69.8% after glucose addition, respectively, which were similar to that of the wild type (Table 2). Maltose and maltodextrin treatments showed similar results. However, the biocontrol effects of the mutant strains did not change significantly after adding only exogenous starch, but the biocontrol level of the wild type could be reached after adding exogenous starch and either AmyS protein or α-amylase. These findings indicated that starch hydrolysate could contribute to the biological control of *Bacillus cereus* 0–9 on wheat sharp eyespot.

**Table 2:** Biocontrol abilities of amylohydrolysis products on wheat sharp eyespot

Treatment	% Inhibition of *R. cerealis* by the strains			
	**B. cereus 0–9**	**ΔamyS**	**ΔamySec**	**ΔamyS/amyS**
negative control	34.3 ± 2.34%	35.9 ± 1.15%	32.8 ± 0.97%	33.6 ± 2.09%
positive control	84.7 ± 1.32%	43.8 ± 2.21%	46.8 ± 0.75%	82.7 ± 1.12%
exogenous starch	83.4 ± 0.32%	40.3 ± 0.91%	41.5 ± 1.75%	79.2 ± 0.66%
exogenous starch and AmyS protein (inactivation)	82.7 ± 0.22%	42.1 ± 0.72%	41.9 ± 0.57%	78.7 ± 0.42%
exogenous starch and AmyS protein ( 1.5 U/mL)	84.6 ± 0.23%	46.3 ± 1.21%	47.5 ± 1.35%	82.2 ± 0.35%
exogenous starch and AmyS protein ( 3.1 U/mL)	85.8 ± 0.37%	57.9 ± 1.47%	59.2 ± 1.94%	85.7 ± 0.87%
exogenous starch and AmyS protein ( 5.8 U/mL)	87.9 ± 0.23%	66.8 ± 1.26%	71.2 ± 1.12%	87.2 ± 0.45%
exogenous starch and AmyS protein ( 10.9 U/mL)	89.6 ± 0.39%	73.5 ± 0.41%	74.2 ± 0.64%	88.6 ± 1.22%
exogenous starch and commercial α- Amylase ( 10.2 U/mL)	88.5 ± 1.82%	72.9 ± 1.55%	73.7 ± 0.96%	86.8 ± 1.06%
exogenous glucose	87.9 ± 0.85%	72.3 ± 1.71%	69.8 ± 1.24%	85.3 ± 0.44%
exogenous maltose	86.1 ± 0.57%	71.9 ± 0.64%	70.2 ± 0.59%	85.7 ± 0.67%
exogenous malto-dextrin	86.7 ± 0.42%	69.3 ± 0.55%	70.7 ± 0.81%	84.4 ± 0.56%

## Discussion

Bacteria can produce a large number of extracellular enzymes to improve their adaptability to the environment [18, 19]. Most of the studies focused on chitinase and cellulase activities, which can degrade fungal cell walls [44–46]. Unlike chitinases, amylases digest starch, and their role in bacterial colonization of roots has rarely been reported [47, 48]. Currently, the research of amylase mainly focuses on industrial production and promoting the absorption and utilization of starch in animals. Satoh E et al. found that the bacterium *Streptococcus bovis* in the rumen of animals has very strong raw starch adsorption and degradation abilities due to the expression of the gene that produces extracellular amylase, which can improve the digestion ability of animals [49]. Here, we studied the involvement of the extracellular amylase gene *amyS* in the colonization ability of *B. cereus* 0–9. In *B. cereus* 0–9, the AmyS protein hydrolyses starch when it is secreted outside the cell, and its secretion depends on the signal peptide located at the 1–27 position. Zhang et al. (2014) found that in *Rhizobiumetli* CFN42, the protein is secreted to the outside of the cell by the signal peptide, which can participate in the nodule signal transduction pathway and play an important role in the symbiotic nodulation process of rhizobia [50]. Yao et al. (2021) also found that *B. subtilis* WS9 can efficiently produce α-amylase by enhancing the signal peptide SPRpmG, which is 2.9-fold greater than the original strain [51]. Yao et al. (2019) also considered that the accumulation of *B. subtilis* extracellular amylase can be enhanced through signal peptide optimization [52]*.*

*B. cereus* 0–9 is a bacterium isolated from wheat roots that has inhibitory effects on *R. cerealis*. To validate that AmyS is involved in the biocontrol ability of *B. cereus* 0–9, we determined the activities of the tested strains against *R. cerealis.* The results showed that the *amyS* gene and AmyS protein can participate in the biological control of wheat sheath blight by *B. cereus* 0–9. There are many factors that influence the effectiveness of bacterial biocontrol, and the ability to effectively colonize plants is a crucial factor. For this reason, we constructed RFP-labeled strains and determined their colonization abilities. We found that the colonization capacities of the Δ*amyS* (RFP) and Δ*amyS*ec (RFP) strains in wheat roots decreased, indicating that AmyS influences the colonization of *B. cereus* 0–9 in wheat roots (Fig. 5). Meanwhile, the complemented strain had the phenotype of WT partially restored, which we speculate is due to the instability of the recombinant plasmid in complemented strain [53, 54]. The recombinant plasmid have the problem of easy loss, because the genes encoded by them will increase the metabolic burden of the cells themselves. Therefore, the antibiotic stress plasmid vector system, the chromosome-plasmid balance killing system of nutrient selection markers and the post segregational killing system (PSK) of the plasmid are often used to stabilize the plasmid [55]. The stabilization of plasmid pAD123-Pgal requires the antibiotic chloramphenicol. In the colonization assay experiment, although chloramphenicol antibiotic was added to the soil, the change of soil moisture during the growth of wheat would cause the concentration of antibiotic to decrease, and the plasmid in some complemented strain would be lost, which showed different result from WT strain. Bulletal. (1991) studied the relationship between the colonization of the biocontrol strain *Pseudomonas fluorescens* 2–79 on wheat roots and the number of wheat disease spots, and proved that the greater the colonization of the biocontrol strain 2–79, the smaller and less numerous spots were produced. When the root colonization amount reached 10^7^–10^8^ CFU/cm, almost no lesions were produced [56]. Li et al. (2006) also reported that when the colonization of *B. subtilis* B47 in the roots and stems of tomato plants reached 10^4^ CFU/g, its control effect on tomato bacterial wilt could reach 79.79% [57]. This is consistent with our results that after the deletion of the *amyS* gene, the reduced biocontrol effect of the mutant strain is related to the ability of the strain to colonize wheat roots.

Endophytes can continuously and stably multiply in plants through competition for nutrients and physical and biological sites to prevent the invasion of other pathogens [58, 59]. Unlike chitinase and cellulase, the extracellular amylase secreted by bacteria cannot destroy the cell walls of plant pathogenic bacteria and affect the colonization ability of bacteria in plant roots [44]. In the interaction between many plants and bacteria, extracellular sugars play an important role, and they participate in the process of bacterial adhesion to the root surface and colonization in the roots [60]. Santaella et al. (2008) proved that extracellular sugars are necessary for the root colonization of the rhizosphere bacterium *Rhizobium sp*. YAS34 in arabidopsis and rapeseed [61]. It is known that amylase can hydrolyze starch to obtain nutrients such as glucose, maltose and maltodextrin, which can meet the growth needs of bacteria [20]. Accordingly, we speculated that the reduced colonization of *amyS* mutant may be related to the nutrients utilization of starch hydrolysis, so we measured the changes of bacterial colonization ability after adding starch hydrolyzates such as glucose, maltose and maltodextrin. Our research showed that the addition of extracellular amylase and starch promoted the colonization of the *amyS* mutant in wheat roots. Adding glucose, maltose, and maltodextrin also showed the same results (Fig. 5). This indicated that the extracellular amylase produced by strain 0–9 can also affect its colonization in wheat roots by hydrolyzing starch to produce glucose and other substances. Our previous work showed that the *psep* gene can participate in the transport of extracellular polysaccharides, affect the formation of biofilms, reduce the resistance of bacteria to environmental stress, and lead to a decrease in colonization ability [62]. Although AmyS can decompose starch to produce monosaccharides such as glucose, it is unknown which proteins are involved in glucose uptake and exopolysaccharide synthesis in *B. cereus* 0–9. At present, there have been many reports on such genes in *B. subtilis*, such as the gene encoding hexose phosphate mutase *yhxB*, the gene encoding glycosyltransferase *yveQ* and the capsular polysaccharide synthesis gene *yveR*, which are involved in the formation of extracellular polysaccharides [63, 64]. Whether these genes exist in *B. cereus* 0–9 and their functions are the direction of our next research.

## Conclusion

In this study, we found that three genes annotated as amylase in the *B. cereus* 0–9 genome. Only after the deletion of the *amyS* gene, the strain lost the ability to produce extracellular amylase, and the signal peptide sequence in the *amyS* gene was related to the extracellular secretion of amylase. The *amyS* gene was closely related to the colonization ability of *B. cereus* 0–9 in wheat roots and the biocontrol ability of *B. cereus* 0–9 to fight against *R. cerealis*, which is the pathogenic fungus of wheat sharp eyespot. Deletion of the *amyS* gene resulted in decreased bacterial colonization and biological control in wheat roots, and this change was caused by a reduction in starch hydrolyzates available to bacteria, such as glucose, maltose, and maltodextrin. In conclusion, our fndings provide a new idea for the effective prevention and treatment of sharp eyespot in wheat.

## Supplementary Information

**Additional file 1: Fig. S1. **Analysisthe amyC gene (FRY47_05825) signal peptide and smart domain 4 (A) Analysis theAmyC protein signal peptide through SignalP 4.1. S-score: Each 5 amino acidcorresponds to 1 S value, with a high S value in the signal peptide region. 6C-score: Each amino acid will have a C value, with the highest C value at theshear 7 site. Y-score: Y value is a parameter considering S value and C value,which is 8 accurate than C value alone, because there may be more than onehigher C value in a 9 series, but only one shear site; the shear site is withsteep S value and high C value. 10 (B) Analysis the AmyC protein smart domainthrough SMART software. The 60-330 11 position is catalytic activity, and thereare two transmembrane areas at each end of the 12 active area. **Fig. S2.** Analysis the amyP gene(FRY47_27030) signal peptide and smart domain. 15 (A) Analysis the AmyP proteinsignal peptide through SignalP 4.1. S-score: Each 16 amino acid corresponds to1 S value, with a high S value in the signal peptide region. 17 C-score: Eachamino acid will have a C value, with the highest C value at the shear 18 site.Y-score: Y value is a parameter considering S value and C value, which is 19accurate than C value alone, because there may be more than one higher C valuein a 20 series, but only one shear site; the shear site is with steep S valueand high C value. 21 (B) Analysis the AmyP protein smart domain through SMARTsoftware. The 23 to 22 401 is the catalytic activity, 413 to 496 is the Cdomain of Aamy, 501 to 577 is the 23 TIG domain, 585 to 681 is the domain ofCBM 20, which has starch binding function. **Fig.S3.** No strains colonization on wheat root (control treatment). **Fig. S4.** Induced expression of amySgene in E.coli BL21(DE3) strain. The strain was cultured to 10 5 31 cfu/mL andsupplemented with 100 μmol/L IPTG for induction culture 32 at 22°C for 6 h.Then subsequently centrifugated and subjected to ultrasonication. 20 33 mM imidazolesolution was used for a Ni column purification, and obtained the 34 purifiedprotein. (a) represent the commercial α-amylase(Ruibio, Germany); (b) 35represent unpurified AmyS protein; (c) represent purified AmyS protein; (d)represent 36 the purified protein was diluted twice-fold; (e) represent thepurified protein was 37 diluted three-fold; (f) represent the purified proteinwas diluted four-fold; (g) 38 represent the purified protein was dilutedfive-fold; (h) represent the purified protein 39 was diluted six-fold. **Fig. S5.** Determination of theinhibitory effect of bacteria and mutant strains on R. 42 cerealis on agarplates. The strains were inoculated in liquid LB medium, cultured at 30 °C to10 8 43 cfu/mL, collected, and washed twice with PBS. Then, an equal volume of44 sterile water was added to the pellet, and 5 μL of bacterial suspension washarvested, 45 inoculated on PDA solid medium, cultured at 30 °C for 3 d. Arepresent the strain 0-9; 46 B represent the strain ∆amyS ; C represent the strain∆amySec; D represent the strain 47 ∆amyS/amyS;, **Table S1.** The primers used in this study.

## Data Availability

Data on the genomes of *B. cereus* 0–9 have been submitted to the NCBI GenBank, which is publicly available, with the GenBank ID CP042874.1 and CP042875.1. The website are as follows: https://www.ncbi.nlm.nih.gov/nuccore/CP042874.1/, https://www.ncbi.nlm.nih.gov/nuccore/CP042875.1/. All the other data generated during this study are included in this published article [and its supplementary information files].
