# Supplementary material for: Production of extracellular amylase contributes to the colonization of Bacillus cereus 0–9 in wheat roots
Source: BMC Microbiol. 2022 Aug 22;22:205. doi: 10.1186/s12866-022-02618-7 (PMC9394064; doi:10.1186/s12866-022-02618-7)
Supplement: Supplementary file 1 — Additional file 1: Fig. S1. Analysisthe amyC gene (FRY47_05825) signal peptide and smart domain 4 (A) Analysis theAmyC protein signal peptide through SignalP 4.1. S-score: Each 5 amino acidcorresponds to 1 S value, with a high S value in the signal peptide region. 6C-score: Each amino acid will have a C value, with the highest C value at theshear 7 site. Y-score: Y value is a parameter considering S value and C value,which is 8 accurate than C value alone, because there may be more than onehigher C value in a 9 series, but only one shear site; the shear site is withsteep S value and high C value. 10 (B) Analysis the AmyC protein smart domainthrough SMART software. The 60-330 11 position is catalytic activity, and thereare two transmembrane areas at each end of the 12 active area. Fig. S2. Analysis the amyP gene(FRY47_27030) signal peptide and smart domain. 15 (A) Analysis the AmyP proteinsignal peptide through SignalP 4.1. S-score: Each 16 amino acid corresponds to1 S value, with a high S value in the signal peptide region. 17 C-score: Eachamino acid will have a C value, with the highest C value at the shear 18 site.Y-score: Y value is a parameter considering S value and C value, which is 19accurate than C value alone, because there may be more than one higher C valuein a 20 series, but only one shear site; the shear site is with steep S valueand high C value. 21 (B) Analysis the AmyP protein smart domain through SMARTsoftware. The 23 to 22 401 is the catalytic activity, 413 to 496 is the Cdomain of Aamy, 501 to 577 is the 23 TIG domain, 585 to 681 is the domain ofCBM 20, which has starch binding function. Fig.S3. No strains colonization on wheat root (control treatment). Fig. S4. Induced expression of amySgene in E.coli BL21(DE3) strain. The strain was cultured to 10 5 31 cfu/mL andsupplemented with 100 μmol/L IPTG for induction culture 32 at 22°C for 6 h.Then subsequently centrifugated and subjected to ultrasonication. 20 33 mM imidazolesolution was used for a Ni [file 12866_2022_2618_MOESM1_ESM.pdf]

Supplementary information

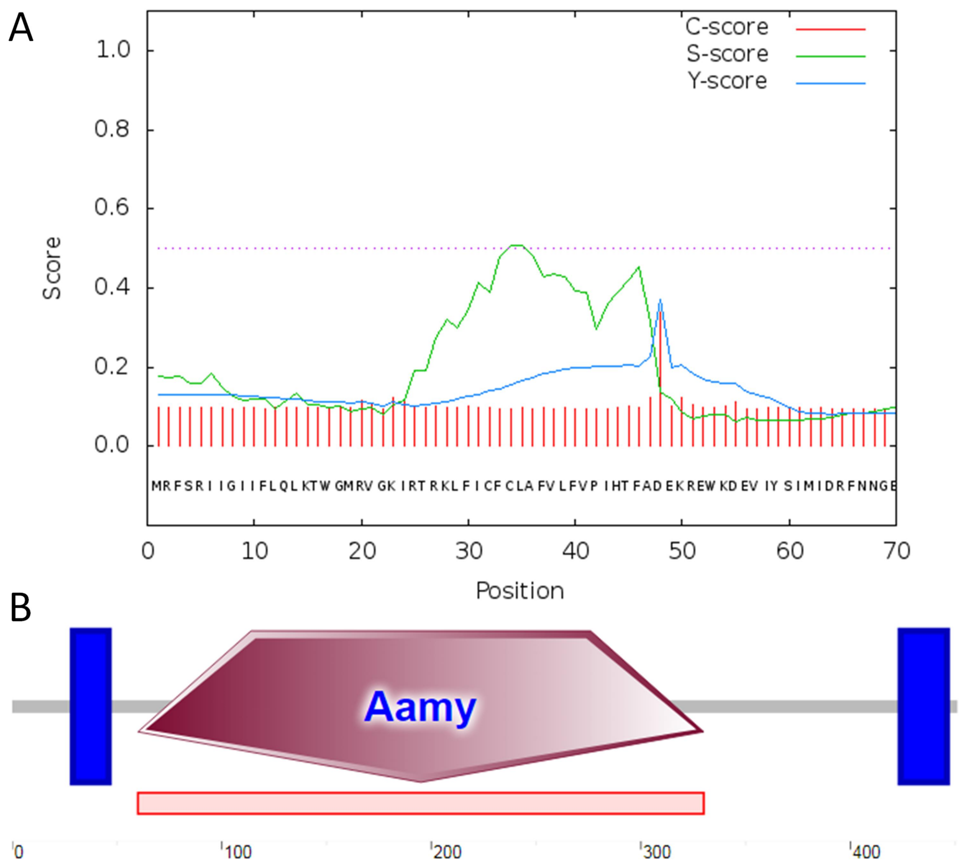

Fig. S1: Analysis the *amyC* gene (FRY47_05825) signal peptide and smart domain (A) Analysis the AmyC protein signal peptide through SignalP 4.1. S-score: Each amino acid corresponds to 1 S value, with a high S value in the signal peptide region. C-score: Each amino acid will have a C value, with the highest C value at the shear site. Y-score: Y value is a parameter considering S value and C value, which is accurate than C value alone, because there may be more than one higher C value in a series, but only one shear site; the shear site is with steep S value and high C value. (B) Analysis the AmyC protein smart domain through SMART software. The 60-330 position is catalytic activity, and there are two transmembrane areas at each end of the active area.

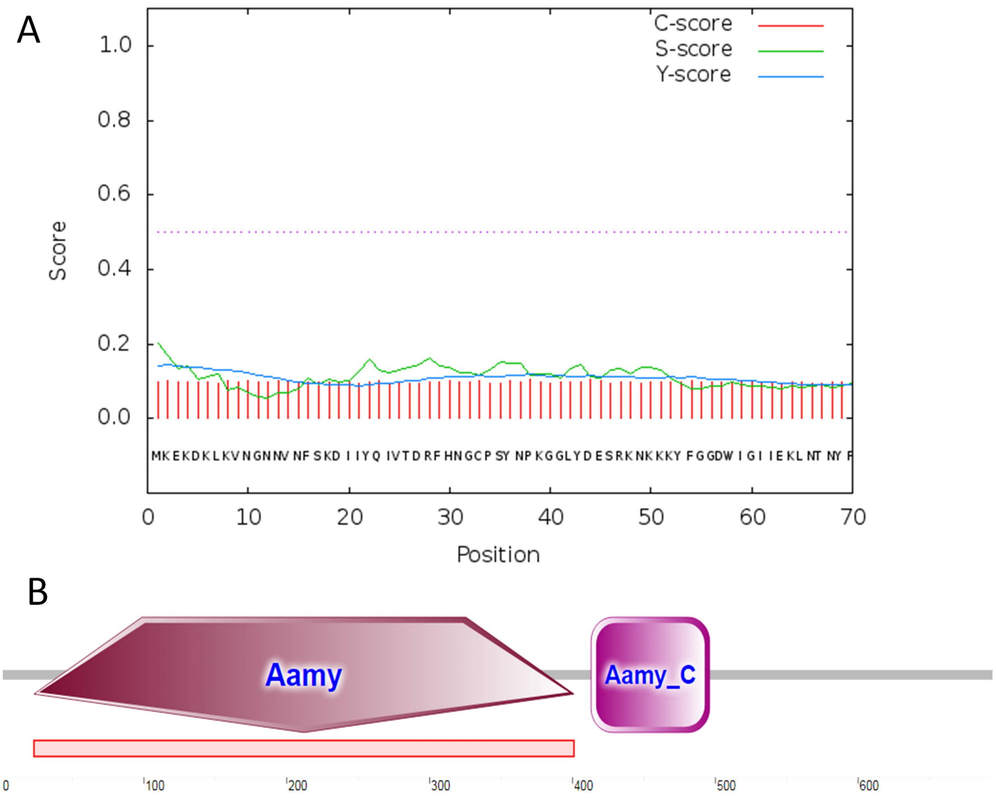

Fig. S2: Analysis the *amyP* gene (FRY47_27030) signal peptide and smart domain. (A) Analysis the AmyP protein signal peptide through SignalP 4.1. S-score: Each amino acid corresponds to 1 S value, with a high S value in the signal peptide region. C-score: Each amino acid will have a C value, with the highest C value at the shear site. Y-score: Y value is a parameter considering S value and C value, which is accurate than C value alone, because there may be more than one higher C value in a series, but only one shear site; the shear site is with steep S value and high C value. (B) Analysis the AmyP protein smart domain through SMART software. The 23 to 401 is the catalytic activity, 413 to 496 is the C domain of Aamy, 501 to 577 is the TIG domain, 585 to 681 is the domain of CBM 20, which has starch binding function.

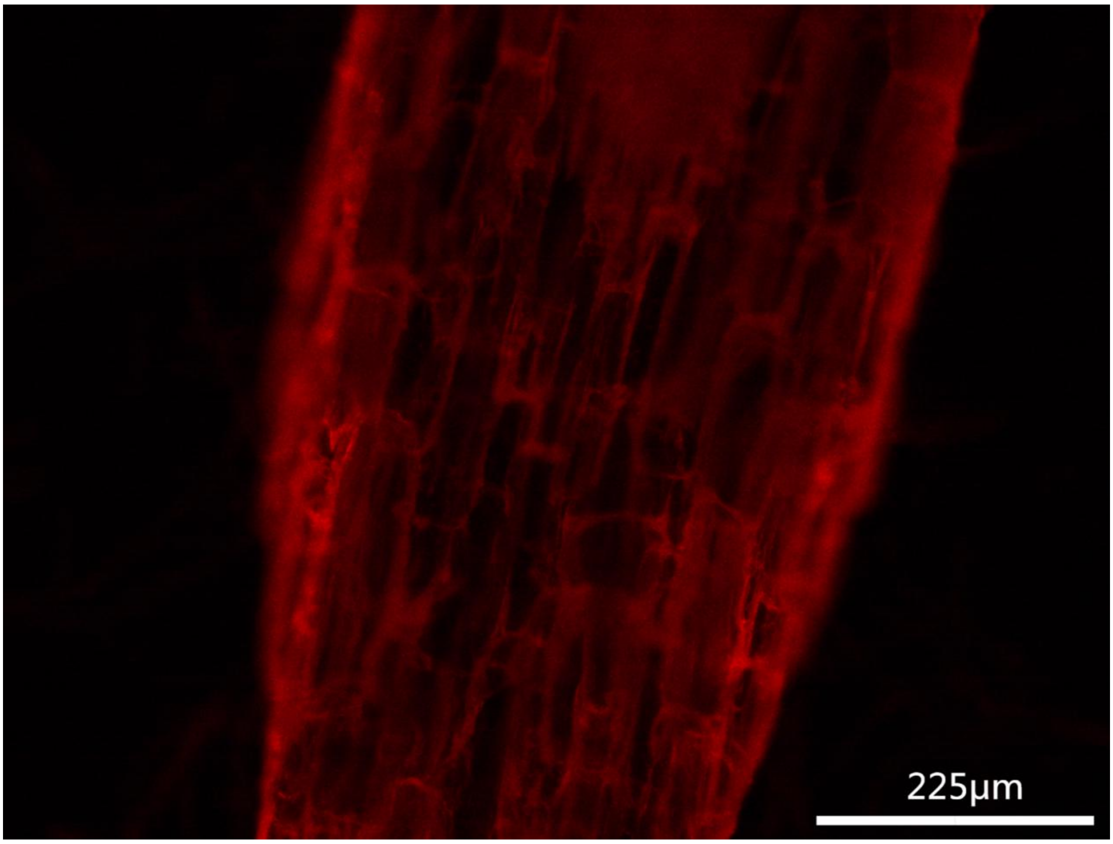

Fig. S3: No strains colonization on wheat root (control treatment)

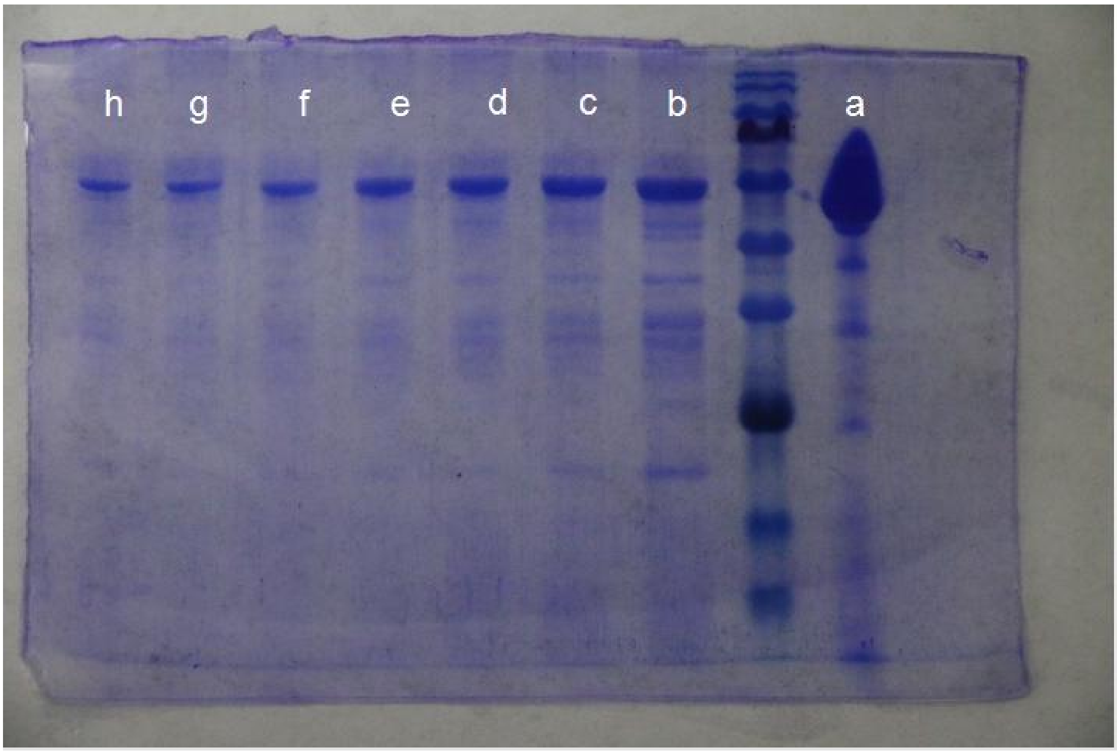

Fig. S4: Induced expression of *amyS* gene in *E.coli* BL21(DE3) strain. The strain was cultured to 10^5 cfu/mL and supplemented with 100 μmol/L IPTG for induction culture at 22°C for 6 h. Then subsequently centrifugated and subjected to ultrasonication. 20 mM imidazole solution was used for a Ni column purification, and obtained the purified protein. (a) represent the commercial α-amylase(Ruibio, Germany); (b) represent unpurified AmyS protein; (c) represent purified AmyS protein; (d) represent the purified protein was diluted twice-fold; (e) represent the purified protein was diluted three-fold; (f) represent the purified protein was diluted four-fold; (g) represent the purified protein was diluted five-fold; (h) represent the purified protein was diluted six-fold.

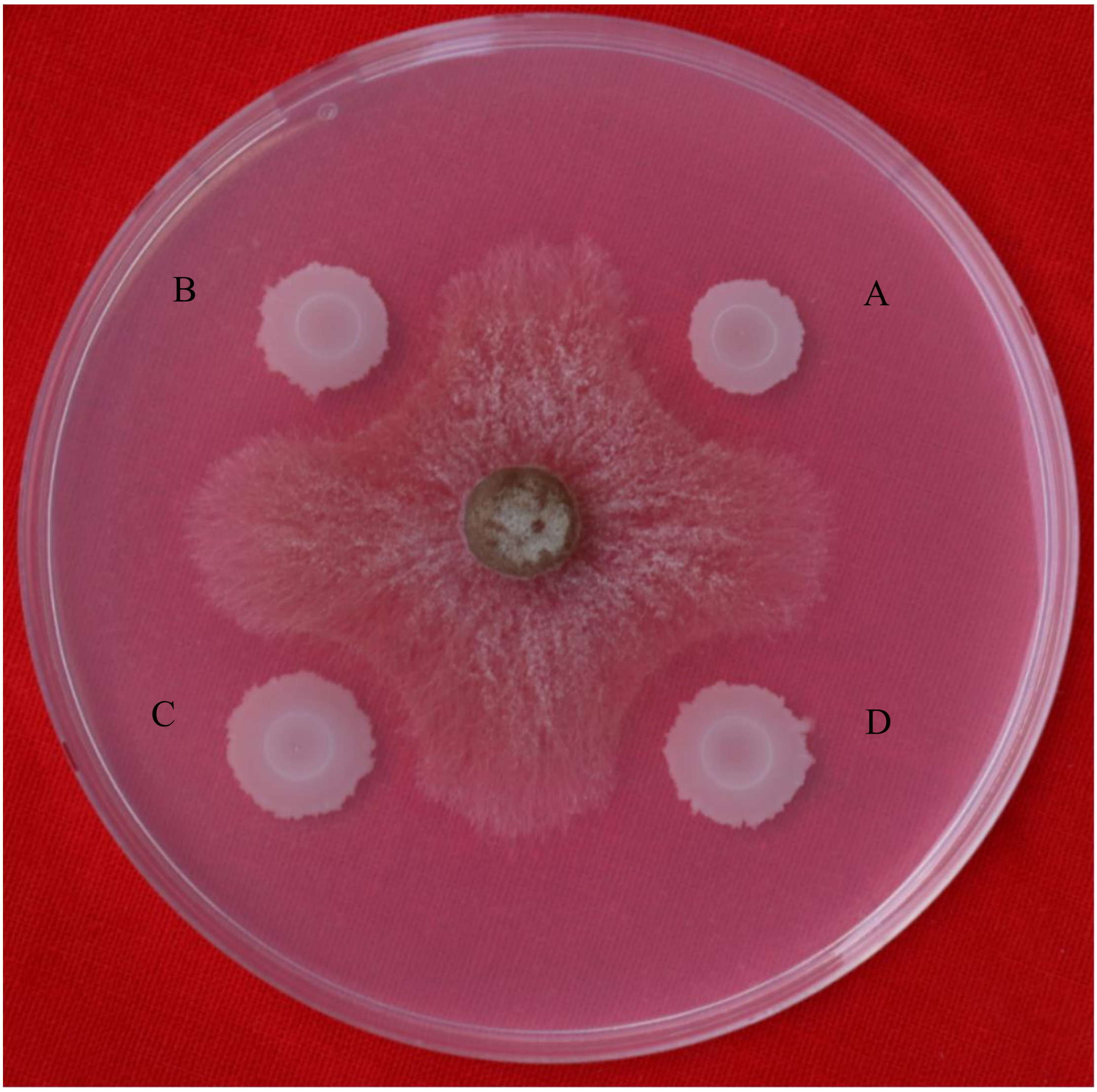

Fig. S5: Determination of the inhibitory effect of bacteria and mutant strains on *R. cerealis* on agar plates. The strains were inoculated in liquid LB medium, cultured at 30 °C to 10^8 cfu/mL, collected, and washed twice with PBS. Then, an equal volume of sterile water was added to the pellet, and 5 μL of bacterial suspension was harvested, inoculated on PDA solid medium, cultured at 30 °C for 3 d. A represent the strain 0-9; B represent the strain Δ*amyS* ; C represent the strain Δ*amyS*ec; D represent the strain Δ*amyS/amyS*;

Table S1: The primers used in this study

Primer name	Alignment (5'→3')
amyC-up-*Bam*HI-s	ACACGGATCCTAAATGCAGTTAAAGCTACGT
amyC-up-*Eco*RI-a	CACAGAATTCTAATTGTAAGAATATTATACCA
amyC-down-*Eco*RI-s	ACACGAATTCTTTCTAATTGCAGTTAAAAGGA
amyC-down-*Xho*I-a	CACACTCGAGTATTAATACTTTTTTCATATTC
amyP-up-*Bam*HI-s	ACACGGATCCTTGTAAAATAGATATATTGATA
amyP-up-*Eco*RI-a	CACAGAATTCAATTAACGTTGTTCCCATTCAC
amyP-down-*Eco*RI-s	ACACGAATTCAGTTGTAAAATGGCAAACTGCA
amyP-down-*Xho*I-a	CACACTCGAGTCAATAAAGTTTCTGCAATATC
amyS-up-*Bam*HI-s	ACACGGATCCACCATCAATTCCACCATTTACA
amyS-up-*Hin*DIII-a	CACAAAGCTTGATCAGTTTCCATATATGTTCA
amyS-down-*Hin*DIII-s	ACACAAGCTTTGTTATTCTTTTAAACATCTG
amyS-down-*Eco*RI-a	CACAGAATTCTCAGTTAGTTTTACAATAAGAG
P*amyS*-*Bam*HI-s	ACACGGATCCCCATTGCGTACATAATGTTAGATA
P*amyS*-amyS-a	TATGCTTTACTCCCCTCATACATCTGTACACCATCCCCTTCCAAG

amyS-PamyS-s	AAGGGGATGGTGTACAGATGTATGAGGGGAGTAAAGCATATG
amyS-*Xho*I-a	CACACTCGAGTTATTGTTGAACATATATGGAAACTGATC
amySec-*Bam*HI-s	ACACGGATCCATGTTTAAAAGAATAACAATAGTC
amyS-*Xho*I-a	CACACTCGAGTTATTGTTGAACATATATGGAAACTGATC
pET28a-*amyS*-*Bam*HI-s	ACACGGATCCATGTATGAGGGGAGTAAAGCATATG
amyS-*Xho*I-a	CACACTCGAGTTATTGTTGAACATATATGGAAACTGATC
